# Hospital Festivals and Meetings

**Published:** 1895-02-23

**Authors:** 


### HOSPITAL FESTIVALS AND MEETINGS.

### ST. THOMAS'S HOSPITAL.

The public meeting in aid of the special appeal fund for St. Thomas's Hospital, which was held in the Egyptian Hall of the Mansion House on Wednesday, February 13th, was well attended. The Lord Mayor was unable to be present in consequence of illness, and in his absence the chair was taken by Sir STUART KNILL. Amongst those on the platform were His Royal Highness the Duke of Connaught (president of the hospital), the Earl of Leven and Melville, the Bishop of Southwark, Sir W. MacCormac, Sir H. Doulton, the Rev. Newman Hall, the Rev. D. McEwan, Mr. T. A. Bevan, and Mr. J. G. Wainwright (treasurer). Letters of regret were received from, amongst others, the Archbishop of Canterbury, the Bishop of Rochester, the Duke of Rutland, the Duke of Devonshire, Lord Thring, Lord Rothschild, Miss Florence Nightingale, and Mr. Burdett-Coutts, M.P. Many warm expressions of sympathy were read from these absent friends. The Duke of Devonshire sent 100 guineas towards the fund. Miss Florence Nightingale in expressing the heartfelt interest she must always feel towards the hospital with whose nursing school she had been so intimately connected for 35 years, sent a contribution of £100, wishing it were "ten times more," and a letter enclosing 5s. from a working man at Folkestone, "who had been kindly treated at St. Thomas's 28 years ago" elicited much applause. A further sum of 10 guineas was also received from Mr. Thomas Creasy.

The CHAIRMAN spoke of St. Thomas's Hospital as belonging in a special sense to the citizens of London. He thought it was their duty to take the initiative at the present crisis in restoring it to its former prosperous position. He looked upon the presence of the Duke of Connaught as an augury of success for their endeavours on its behalf.

The Duke of CONNAUGHT then moved the following resolution: "That this meeting has heard with sincere regret that notwithstanding the large increase in the population of South London, and the consequent greater demand for hospital treatment, there are several wards in St. Thomas's Hospital lying empty and unused owing to the income of the charity not proving sufficient to maintain a greater number of beds than those now in use (436)." His Royal Highness went on to speak of the past history of the hospital, one of the oldest in London granted by a charter of Edward VI. to the Mayor and citizens of London to be a hospital for the City. He knew it was said by some people with regard to the present very fine structure, "Oh! we will not support hospitals which have gone to such enormous expense; they have followed the fads of the doctors." That must be a matter of opinion, but what might not have been said, had a hospital, built so recently as 1872, not been a modern structure containing all the requirements of a modern hospital? The income of St. Thomas's Hospital had dwindled largely, owing to agricultural depression, and the hospital had been heavily hit by the increase in rates, it having been decided on appeal by the House of Lords that it could not be exempt from that burden. He wished particularly to draw attention to the fact that this hospital was free in every sense of the word, a governor's signature had no weight unless a case were truly deserving The population round the hospital had increased from 644,000 in 1871 to 961,000 in 1891, and was now upwards of 1,000,000. The in-patients during the past year numbered 5,840, while there were 24,082 out-patients and 82,782 casualty patients who received treatment. He hoped the public might be confidently looked to to support an institution which is capable of doing so vast an amount of good. English people were proud to think that all their great charities were supported by voluntary contributions, so different from the system pursued abroad. He appealed warmly to the public, and he hoped the appeal would not be without success, to come forward and support one of the most deserving institutions in this country.

The Bishop of SOUTHWARK, in the absence of the Bishop of Rochester, seconded the resolution, and urged the importance of having these empty wards filled before subscribing to schemes for building hospitals elsewhere. One or two great centres of hospital work were better than several small ones, both on economical grounds and on account of the higher class of skill obtainable.

Sir WILLIAM MACCORMAC spoke of the importance of the work of the Medical School, which he considered one of the most perfect in the kingdom, and of the mutual dependence upon each other of school and hospital.

Other speeches followed by the Rev. NEWMAN HALL and the Earl of LEVEN and MELVILLE, who moved the second resolution: "That taking into consideration the noble work accomplished by the hospital since its foundation in 1551, both as a medical charity and also as one of the leading schools of medicine and surgery in the kingdom, this meeting views with considerable alarm the falling off of its income from country estates, and records its opinion that an effort should be made to obtain the sum of £100,000 with the view of enabling the at present closed wards to be opened for the free treatment of the poor of this great metropolis." The Rev. D. MCEWAN and Mr. BEVAN also spoke. Finally Mr. WAINWRIGHT put a brief statement of certain financial facts before the meeting, explaining that, owing to the continued depression in landed property, farms which some years ago brought in £1,270 a year, were now let for £385, and dwelling especially on the enormous sum, £2,500 a year which the hospital had to contribute to the parish rates.

It was also resolved to make a special appeal to the great City guilds for their help, and before the meeting finally closed Sir Stuart Knill announced that Her Majesty the Queen had been pleased to contribute £100 towards the fund.

Since this meeting we understand that the Duke of Connaught has contributed £100, and that Messrs. Barclay, Perkins have contributed £1,000 towards the St. Thomas's Hospital Special Appeal Fund.

### NATIONAL HOSPITAL FOR CONSUMPTION, VENTNOR.

The annual meeting of the governors of this hospital took place on Thursday, February 14th. In the absence of Sir Richard Webster the chair was taken by Sir Algernon Borthwick. The report showed a satisfactory increase in income, chiefly due to a gift of £1,500, part of £2,000 promised by an anonymous donor. The receipts for 1894 amounted to £12,268, as against £10,091 for the preceding year, the expenditure being £12,011. During the year the hospital has sustained a serious loss by the death of its founder, Dr. Arthur Hill Hassell. Mr. Frederick C. Colman had, owing to ill-health, resigned the office of treasurer, and his son had been appointed in his stead. The physician to the hospital, Dr. J. G. Sinclair Coghill gave an excellent account of the medical work during the past year. The death-rate had been only 3 per cent., and the beneficial results chronicled had increased. The Chairman commented on the report as being one of the most satisfactory he had ever known. He hoped the public, when they knew what had been the hospital's work for the past twenty-five years, would come forward and support it as liberally as it deserved. He remembered the day when the case of anyone attacked with consumption was considered hopeless, but this hospital had proved that the disease might be successfully fought. At the close of the meeting Dr. Sinclair Coghill presented to the hospital, in the name of the subscribers (members of the staff and others), a life-size portrait in oils of Mr. Frederick C. Colman, speaking of the warm interest the latter had ever shown in the affairs of the institution.

### CITY OF LONDON HOSPITAL FOR DISEASES OF THE CHEST, VICTORIA PARK, E.

The annual meeting of the governors was held on the 14th inst. at the hospital under the presidency of Mr. Julian Hill, treasurer. The report referred to the fire which had taken place at the hospital in the early part of last year. Steps had been taken for the prompt repair of the damage caused by it, and the hospital had for some time past been again in full working order. Certain wards had, however, been thrown out of use for a time, and others had been temporarily closed during the renewal of the corridor floors. The number of in-patients received, viz., 937, had. for these reasons, been less than in the previous year, but 16,054 out-patients had been relieved, the year having been in this respect one of exceptional activity. Although the income showed an increase on that of 1893, it had been inadequate to meet the expenditure, and the committee had been obliged to borrow £2,500, making, with the undischarged loan of the previous year, an indebtedness of £4,000, and they had also to draw upon the small reserve by selling £1,000 Consols. The Chairman, in proposing the adoption of the report, stated that since its preparation the receipt of a timely bequest had enabled the committee to repay the loan. He drew attention to the fact that in restoring the damage done by the fire, advantage had been taken of the opportunity to increase and improve the nurses' dormitories, and that separate sleeping accommodation had been provided for each day and night nurse. A much-needed reconstruction of the sanitary appliances in the south wing had also been effected. The hospital was now in a thoroughly efficient state, and the chairman earnestly asked for public support to enable its good work to be carried on. It was notified that the office of the institution would be removed from the City to the hospital at midsummer next, a change which, the committee were convinced, would promote efficiency. Alterations were made in the bye-laws to provide that in future the Medical Committee shall consist of all the members of the medical staff; that the audit of the accounts shall be entrusted to chartered accountants elected at annual court; and that the privileges of firms and companies, who have not named a member to receive them, shall continue for a period not exceeding twenty years from date of motion being made. The committee and auditors were elected for the ensuing year. A vote of thanks to medical staff, committee, and chairman closed the proceedings.

